# Track Surfaces Used for Ridden Workouts and Alternatives to Ridden Exercise for Thoroughbred Horses in Race Training

**DOI:** 10.3390/ani8120221

**Published:** 2018-11-26

**Authors:** Ashleigh V. Morrice-West, Peta L. Hitchens, Elizabeth A. Walmsley, R. Chris Whitton

**Affiliations:** U-Vet Equine Centre, Melbourne Veterinary School, University of Melbourne, 250 Princes Hwy, Werribee VIC 3030, Australia; a.morrice@student.unimelb.edu.au (A.V.M.-W.); peta.hitchens@unimelb.edu.au (P.L.H.); eawa@unimelb.edu.au (E.A.W.)

**Keywords:** Thoroughbred, racehorse, equine, training, exercise, surface, track

## Abstract

**Simple Summary:**

Musculoskeletal injury rates for Thoroughbred racehorses in training and racing differ between racing jurisdictions. The aetiology of these injuries is multifactorial, but one potentially important and modifiable risk factor is the track surface on which horses train. However, the extent to which different track surfaces are used by trainers has not been clearly established. Similarly, the extent of use of alternatives to ridden exercise between different jurisdictions is unknown. Trainers in Victoria, Australia, use a combination of turf, sand, synthetic and dirt training track surfaces. Sand or synthetic surfaces were most commonly used for slow workouts and turf or synthetic tracks for fast workouts. A high proportion of trainers raced horses on surfaces that were not regularly used for training, and 89% of trainers used alternative exercise methods in addition to overground ridden workouts. Determining types of surfaces and alternatives to ridden exercise used during training, and to what extent they are used, is the first step in understanding their association with the risk of injury. The future aim is mitigating injury risk by recommending safer track surfaces.

**Abstract:**

Little is known about the types of surfaces used during training of Thoroughbred racehorses or methods of exercise used in addition to ridden track-work. Our aims were to (1) describe the types of surfaces used in the training of Thoroughbred racehorses and to (2) identify alternative approaches used to exercise horses in addition to, or in place of, ridden overground track-work. Information regarding surface and alternative exercise methods was collected as part of an in-person survey of training practices of 66 registered Thoroughbred trainers in Victoria, Australia. Sand and synthetic surfaces were used by 97% and 36% of trainers respectively for slow-workouts, with galloping on turf training tracks used in training regimens by 82% and synthetic by 58% of trainers. Of those trainers utilising turf tracks, only 34% of gallop training was completed on turf despite turf being the predominant racing surface. Almost 90% of trainers used alternatives to ridden exercise. There is substantial variation in training surface used and alternative types of exercise undertaken by Victorian trainers. Future research should focus on how such practices relate to injury risk, particularly as it relates to the importance of musculoskeletal adaptation to specific race-day surfaces.

## 1. Introduction

Two aspects of management that have been identified as potentially modifiable risk factors for injury in Thoroughbred racehorses are the track surface on which horses train and race, and methods of fitness training that avoid galloping on a track [1,2,3,4,5,6]. Despite more fractures occurring in horses when training compared to those on race-days, likely attributable to greater time spent training than racing and therefore greater time at risk, investigations of track surface as a risk factor for musculoskeletal injury (MSI) have predominantly been confined to the surface on which the horse was racing on the day of injury [1,2,3,7,8,9]. Track surface type and condition is consistently a risk factor for race-day MSI, but there is also evidence that training track surface is associated with fracture risk and site of fracture, with high-speed exercise on sand and dirt tracks at higher risk of catastrophic injury compared to turf. [4,10,11,12,13,14,15].

In Australia, racing is predominantly conducted on turf tracks, although in recent years racing on synthetic surfaces has been introduced. In Victoria in the 2016/2017 racing season (1 August 2016 to 31 July 2017), 91.6% (38,150/41,668) of flat race starts were conducted on turf tracks, with the remaining flat race starts conducted on synthetic surfaces [16]. Generally, training tracks in Victoria are turf (grass), sand, synthetic or dirt. Synthetic track surfaces are made up of varying proportions of sand, synthetic fibres and wax or polymeric binder over a hard porous base. Sand tracks are pure sand over a non-porous base, so that they can be kept moist to maintain some binding of the sand particles. Victorian synthetic surfaces are predominately Polytrack (Martin Collins Enterprises Ltd., Australia) and Pro-Ride (Pro Ride Racing Australia Pty Ltd., 2010, VIC, Australia). Dirt tracks are a combination of clay and sand on a hard base. The proportion of different types of surfaces utilised in racehorse training in Australia is unknown, therefore the extent to which horses’ bones are prepared for racing by training on specific surfaces is yet to be established.

A variety of exercise approaches have been proposed as alternatives to ridden track work on a flat surface. Treadmills provide a consistent firm surface without rider weight and are unaffected by climatic conditions with complete control over the speed of the workout [5,17]. Swim training has been suggested to reduce the frequency of limb injuries due to the absence of weight bearing forces [6]. Hill work and incline treadmills reduce the speed of the workout—speed being a factor associated with higher injury risk [18,19].

Despite the potential importance of training surface on injury risk, there are few studies detailing the types of surfaces used during overground racehorse training, or that investigate the impact of alternative exercise methods. The aims of this study were, therefore, to describe the variation in training practices of Thoroughbred trainers in Victoria, Australia as they relate to (1) surface use in slow and fast workouts; and (2) exercise alternatives to overground ridden workouts. Determining types of surfaces and alternatives tor ridden exercise used during training, and to what extent they are used, is the first step in understanding their association with the risk of injury.

## 2. Methods

This study was conducted as a component of a semi-structured survey of training regimens of 66 registered Victorian Thoroughbred trainers [20]. Briefly, the total population of 889 registered trainers were eligible to participate in the study. Participating trainers were a convenience sample according to industry personnel and veterinarian recommendations, followed by random selection from the registry to enlist approximately equal numbers of the three licensing levels according to experience and number of horses, and across the three regional classifications (Metropolitan, Provincial, Country) at all major training centres in Victoria, Australia. All trainers who agreed to participate were interviewed.

Trainers were asked about the typical surfaces utilised by their horses for slow workouts and gallop exercise, the proportion of monthly slow workouts and fast workouts on each surface used, how these surfaces related to the types of surfaces raced on, as well as whether or not they utilised alternative training methods in addition to ridden track-work and the details of their use. 

Data analyses were conducted using Stata/SE version 15.0 (StataCorp. 2017. Stata Statistical Software: Release 15. College Station, TX, USA: StataCorp LP). Data were examined for normality via Shapiro–Wilk tests and histograms. Normally distributed data were reported as mean and standard-deviations and median (IQR) for non-normally distributed data. Count data were reported as counts and percentages. 

Ethics approval was obtained by the University of Melbourne Faculty of Veterinary and Agricultural Sciences’ Human Ethics committee (Reference 1647911.1).

## 3. Results

Surveyed trainers included 19 Class A, 24 General, and 23 Restricted trainers primarily based at 19 Metropolitan, 36 Provincial and 11 Country training tracks. There was wide variation in surfaces on which horses were trained and the proportion of training spent on each surface for both slow workouts (Table 1) and fast workouts (Table 2). Trainers frequently used more than one type of surface and the selection of surface was generally based on the anticipated maximal speed of the workout. Most trainers used sand or synthetic surfaces for slow workouts, with fewer using dirt or turf tracks. Most trainers used turf or synthetic tracks for galloping horses though many utilised both surfaces. Sand was the next most commonly used surface for galloping, whereas dirt tracks were rarely used for galloping horses. Thirty-four trainers provided rationale for their surface use decisions based on the tracks available to them. The most common response was limited access or availability of turf tracks (n = 16), followed by synthetic surfaces being in better condition in wet weather (n = 7) and sand tracks providing less concussion (n = 7), with another two trainers reporting synthetic surfaces as too compacted. Two trainers described basing the decision on which track was best maintained and two described dirt tracks being the most natural surface. One trainer reported that smaller tracks were more appropriate choices for two-year-olds entering work.

The most utilised racing surface in Victoria is turf. Among trainers that utilised turf tracks in training, only 28% (15/54) undertook half or more of their gallop exercise on this surface. For all race-day surfaces (turf and synthetic) 36% (24/66) of trainers only raced horses on surfaces they were regularly exposed to in training (i.e., at least weekly). Thirty-four percent (23/66) of trainers raced horses on surfaces they had previously raced or trained on but were not surfaces used routinely for training (i.e., use of the specific surface only for the occasional gallop in or prior to trialling or racing). The scenario was reported to occur when certain training facilities restricted access to gallop on turf tracks, allowing only those horses that were nominated in specific upcoming races that week. Twenty-nine percent (19/66) of trainers reported that their horses had no prior exposure to the surface used on race day, or only during an occasional preparatory gallop or trial. In addition, 20% (13/66) of trainers used hill work in training, 12% (8/66) exercised horses on firm beach sand, and 9% (6/66) on soft beach sand. 

Eighty-nine per cent of trainers (59/66) used alternate methods of exercise in addition to ridden track-work. Sixty-eight per cent (45/66) regularly swam their horses, 50% (33/66) used a walker and 39% (26/66) a treadmill. Trainers that regularly swam horses did so a median of five times per week (IQR 2.75–6.5), with an additional four trainers only swimming after hard gallops and six only using swimming sporadically. The median time spent by horses on the walker per day was 55 min (IQR 40–60; answered by 28 of the 33 users). Six percent (4/66) used water walking in regular training and an additional 6% (4/66) used water walkers for pre-training or rehabilitation purposes. The majority of treadmill training was at trot or canter, but 10 of the 26 treadmill users (38%) also performed fast workouts. Treadmills were used a median of 1.75 times per week (IQR 1.25–2.0; reported by eight trainers), with nine trainers (35%) reporting only using treadmills on specific horses, four of which were based on horses being hard to handle, four reported using treadmill in circumstances of poor weather or lack of rider availability, and one reported using treadmills especially for fillies (reducing weight carried by lighter horses in workouts). Nine of the 26 trainers used treadmills to replace slow workouts for horses with tendon or soft tissue injuries, and five of these used treadmills for most or all fast workouts other than trialling for this specific group of horses (three of the 10 trainers using treadmills for fast work was exclusively for this group of horses). Treadmill slow work protocols consisted of a median of six minutes trotting (IQR 5–10) and five minutes cantering (IQR 4–5). Of the seven trainers galloping horses on treadmills, two monitored blood lactate concentration during high intensity exercise. Protocols involved one to three fast workout intervals, and inclines of four degrees for walking and trotting and up to six degrees for cantering and faster. Maximal speeds were variable but ranged from 32 kph to 45 kph for one minute intervals on an incline. 

## 4. Discussion

In Victoria, Australia, surveyed trainers’ use of track surfaces for training varied based on the anticipated speed of the exercise session and track availability. All combinations of turf, sand, synthetic and dirt were used, but sand or synthetic surfaces were most commonly used for slow workouts and most trainers galloped horses on turf or synthetic tracks. Approximately one-third of trainers surveyed raced horses on surfaces they were regularly exposed to in training, with the remaining two-thirds racing horses on surfaces they were not accustomed to in their usual monthly training regimens. The majority of trainers employed some form of alternative exercise to overground ridden workouts. 

Turf surfaces were used by 80% of trainers, but only for 34% of each horse’s galloping exercise. In comparison, a cohort from a United Kingdom (UK) study (assessing tibial and pelvic fracture risk) had 81% of horses performing 70% or more of their high-speed work on turf or peat-moss training tracks [13]. Despite a large proportion of high-speed workouts being undertaken on synthetic surfaces by horses of more than half of the surveyed trainers (and up to 100% of gallop exercise in some cases), less than 10% of races and race meetings are conducted on this surface. Synthetic training surfaces in Victoria were used for galloping more than slow-workouts. Comparatively, all-weather (synthetic) surfaces in the UK were predominately used in workouts below high-speed exercise, with 51% of horses completing greater than 70% of total monthly distances on all-weather tracks and the remaining 49% on a combination of all-weather, turf and woodchip [13]. An equally distributed number of the horses presented for scintigraphic examination to a Canadian equine hospital trained on dirt and synthetic training tracks [4]. In our study, almost all trainers had horses undertake a high proportion of slow workouts on sand training tracks, and a third of trainers utilised sand for a large amount of fast workouts whereas less than 10% of trainers employed dirt surfaces for fast work. This is in contrast to a clinical population of horses presenting to a United States of America (USA) equine hospital, where 64% had been trained on dirt training tracks with the remaining 36% training on turf tracks [4]. Exercise of horses on hill tracks was used by 20% of trainers in the present study, whereas UK training regimens more commonly incorporate a greater proportion of hill training [21].

Track surface properties have the potential to influence how loads are generated in the limb and therefore the development of bone fatigue injuries [4,15]. There has been some indication of an association between the use of turf during training gallops and lower odds of lateral condylar third metacarpal/metatarsal fracture [22]. Additionally, all-weather (synthetic) training tracks have been shown to be associated with increased risk of pelvic and tibial stress fractures compared to turf [13]. However, through scintigraphic examination, a higher incidence of stress fractures was identified in horses trained on synthetic compared to dirt surfaces [4]. Galloping exercise on sand and dirt surfaces is associated with higher risk of catastrophic MSI compared to turf-tracks [4,10], and impact force and surface stiffness is reportedly significantly higher for dirt surfaces compared to synthetic surfaces [11,12]. Bone adapts to its loading environment but the importance of skeletal adaptation to galloping on an individual surface is unknown [23]. A high proportion of trainers in the present study trained horses with limited exposure to race-day equivalent surfaces. For example, some trainers completed all high-speed gallop work on sand which is not used as a racetrack surface for Thoroughbreds in Australia. It is therefore possible that these horses’ bones may not be appropriately adapted to the potential forces and surface properties encountered on race-day.

Alternative methods of fitness training to ridden track-work are widely used, as evidenced by the high percentage of the study group utilising such approaches in typical training regimens. The most commonly used alternative exercise was swimming, followed by the use of mechanical walkers. The proportion of trainers using swimming and walkers as part of their typical training regimens in other countries is unknown although both appear commonly used in Australasia [24,25]. A New Zealand study of 14 trainers reported horses first entering racing having a median of one swim day preceding the start of racing and a median of 40 min per day on mechanical walkers, compared to a median of five swim days a week for trainers using swim training and a daily 55 min on mechanical walkers in the present study [25]. More than a third of trainers in the present study utilised treadmills in training. The extent to which this is comparable to other racing jurisdictions is undetermined.

Alternative training methods have been suggested to have potential benefits in mitigating the risk of injury by reducing the cumulative loading from ridden over-ground exercise while maintaining cardiovascular fitness and oxidative muscle capacity [6,26]. High speed treadmill exercise has been shown to generate adaptive bone modelling and one study demonstrated an association with improved racing performance compared with overground exercised horses [5,27]. Comparatively, in the case of swimming, the level of exertion has been suggested to be equivalent to a fast trot or slow canter rather than a gallop replacement, but provides much less skeletal loading for adaptation [28]. There has been little research into the role of walking distance on injury occurrence, but increasing time on mechanical walkers in one study was associated with greater risk of involuntary interruptions to training (both MSI and Dorsal Metacarpal Disease) [29]. 

This study had some limitations. Due to the sampling method and voluntary nature of the study, selection bias may have been present. Trainers with an interest in the outcomes of the study may have been more willing to participate. Surface data were collected at the trainer level (i.e., general practices across his/her stable rather than for specific horses) and therefore the variation by individual horses could not be investigated. Trainers’ access to certain facilities and specific track types varied based on the location. However, many of the surveyed trainers were willing to travel to other training facilities, beach areas, etc., to gain access to surfaces not available locally. 

## 5. Conclusions

This study highlights the variation in surface and alternate training practices used by a sample of Thoroughbred trainers in Victoria, Australia. The importance of skeletal adaptation to galloping on each individual surface is not clear, but given the large degree of variation in use, an understanding of the association between training environment surfaces and the injury risk posed by the respective surface is likely to be important for developing preventative strategies. Future research should focus on how such practices relate to injury risk and the importance of adaptation to specific race-day surfaces. 

## Figures and Tables

**Table 1:** Percentage of Thoroughbred trainers (n = 66) using each surface type for slow workouts, and the mean percentage of workouts spent on each surface used in Victoria, Australia.

Slow Work Surface	Number of Trainers	Percentage of Trainers (%)	Percentage of Work by Surface Type		
			Mean	SD.	Range
Synthetic	23	34.9	35.4	27.6	5, 100
Sand	64	97.0	83.4	25.3	10, 100
Dirt	9	13.6	37.3	32.7	1, 90
Turf	3	4.6	15.3	21.4	1, 40

**Table 2:** Percentage of Thoroughbred trainers (n = 66) using each surface type for fast workouts, and the mean percentage of workouts spent on each surface used in Victoria, Australia.

Fast Work Surface	Number of Trainers	Percentage of Trainers (%)	Percentage of Work by Surface Type		
			Mean	SD.	Range
Synthetic	38	57.6	68.4	27.9	10, 100
Sand	22	33.3	71.3	30.1	5, 100
Dirt	5	7.6	83.0	17.2	60, 100
Turf	54	81.8	34.2	33.7	1, 100
